# NOODAI: a webserver for network-oriented multi-omics data analysis and integration pipeline

**DOI:** 10.1093/bioinformatics/btaf553

**Published:** 2025-10-06

**Authors:** Tiberiu Totu, Rafael Riudavets Puig, Lukas Jonathan Häuser, Mattia Tomasoni, Hella Anna Bolck, Marija Buljan

**Affiliations:** Nanomaterials in Health Laboratory, Swiss Federal Laboratories for Materials Science and Technology (Empa), 9014 St. Gallen, Switzerland; Swiss Institute of Bioinformatics (SIB), 1015 Lausanne, Switzerland; Department of Health Sciences and Technology, Eidgenössische Technische Hochschule Zürich (ETH), 8092 Zurich, Switzerland; Nanomaterials in Health Laboratory, Swiss Federal Laboratories for Materials Science and Technology (Empa), 9014 St. Gallen, Switzerland; Swiss Institute of Bioinformatics (SIB), 1015 Lausanne, Switzerland; Nanomaterials in Health Laboratory, Swiss Federal Laboratories for Materials Science and Technology (Empa), 9014 St. Gallen, Switzerland; Swiss Institute of Bioinformatics (SIB), 1015 Lausanne, Switzerland; Department of Health Sciences and Technology, Eidgenössische Technische Hochschule Zürich (ETH), 8092 Zurich, Switzerland; Department of Ophthalmology, University of Lausanne, Fondation Asile des Aveugles, Jules Gonin Eye Hospital, 1004 Lausanne, Switzerland; Platform for Research in Ocular Imaging, Fondation Asile des Aveugles, Jules Gonin Eye Hospital, 1004 Lausanne, Switzerland; Department of Pathology and Molecular Pathology, University of Zurich and University Hospital Zurich, 8091 Zürich, Switzerland; Centre for AI, School of Engineering, Zurich University of Applied Sciences (ZHAW), 8400 Winterthur, Switzerland; Nanomaterials in Health Laboratory, Swiss Federal Laboratories for Materials Science and Technology (Empa), 9014 St. Gallen, Switzerland; Swiss Institute of Bioinformatics (SIB), 1015 Lausanne, Switzerland

## Abstract

**Summary:**

Omics profiling has proven of great use for unbiased and comprehensive identification of key features that define biological phenotypes and underlie medical conditions. While each omics profile assists characterization of specific molecular components relevant for the studied phenotype, their joint evaluation can offer deeper insights into the overall mechanistic functioning of biological systems. Here, we introduce an approach where, starting from representative traits (e.g. differentially expressed elements) obtained for each omics profile, we construct and analyze joint interaction networks. The resulting networks rely on the existing knowledge of confident interactions among biological entities. We use these maps to identify and describe central elements, which connect multiple entities characteristic of the studied phenotypes and we leverage MONET network decomposition tool in order to highlight functionally connected network modules. In order to enable broad usage of this approach, we developed the NOODAI software platform, which enables integrative omics analysis through a user-friendly interface. The analysis outcomes are presented both as raw output tables as well as informative summary plots and written reports. Since the MONET tool enables the use of algorithms with strong performance in identifying disease-relevant modules, NOODAI software platform can be of a high value for analyzing clinical multi-omics datasets.

**Availability and implementation:**

NOODAI is freely accessible at https://omics-oracle.com. Source code is available under GPL3 at: https://github.com/TotuTiberiu/NOODAI with the DOI: 10.5281/zenodo.17203984.

## 1 Introduction

Omics characterization of biological samples allows for comprehensive identification and quantification of molecules present in the cells. Detection of genome mutations, as well as measurement of abundances of different forms of transcripts, proteins, metabolites and epigenetic changes, have proved to be of great use in understanding cellular processes that define biological phenotypes. Datasets obtained from different omics profiles are often analyzed and interpreted individually. Nevertheless, due to the strong interplay of different types of molecules in exerting biological functions, a simultaneous evaluation of multiple omics profiles can allow for the identification of novel features representative of the system and critical to its function.

Several methods for integrating multiple omics profiles have been proposed in the literature, (Subramanian *et al.* 2020, Picard *et al.* 2021). Among these, MOFA (Argelaguet *et al.* 2018), mixOmics (Rohart *et al.* 2017), and MCIA (Meng *et al.* 2014) are prominent examples of statistical frameworks that integrate normalized measurements from different omics layers and jointly analyze them with the aim of identifying feature combinations (such as gene mutations, transcript and protein levels) that capture a high fraction of variation in molecular phenotypes across conditions. Another frequently used omics integration method is WGCNA (Langfelder and Horvath 2008), which is based on the usage of correlation analysis for data-driven network generation. Other approaches include MCFA (Brown *et al.* 2023), iClusterPlus (Mo *et al.* 2013) and SNF (Wang *et al.* 2014). While of great value, data-driven methods often require solid programming skills and specialized knowledge for interpreting the reported trends.

To enhance the accessibility of methods for the integration of different omics layers several web platforms have been developed. The 3Omics platform offers pathway enrichment and feature co-expression analysis starting from user-provided features of interest (Kuo *et al.* 2013). Two other frequently used platforms are OmicsNet (Zhou *et al.* 2022) and OmicsAnalyst (Zhou *et al.* 2021). OmicsNet relies on previously known interactions for building networks while using as network seeds significant hits from individual omics analyses. OmicsAnalyst supports data-driven analyses and offers multi-view clustering as well as dimensionality reduction based on both supervised (DIABLO from the mixOmics suite) and unsupervised (MOFA, MCIA) methods. Both platforms provide easy to use visualization tools as well as methods for network analysis and functional enrichment assessment. Examples of other webtools for multi-omics analyses include Omics Integrator (Tuncbag *et al.* 2016), GeneTrail2 (Stöckel *et al.* 2016), MAINE (Gruca *et al.* 2022), Mergeomics 2.0 (Ding *et al.* 2021), and PaintOmics4 (Liu *et al.* 2022). The network-based approaches incorporated in these webtools frequently use graph analysis methods that were not specifically designed for biological networks. In general, extracting biologically and clinically relevant insights from multi-omics profiles still presents a considerable challenge.

The most often applied approaches for the analysis of biological networks include node centrality estimates and network decomposition, such as network modularization. A recent community-based Disease Module Identification DREAM Challenge (Choobdar *et al.* 2019) has comprehensively assessed performance of 75 network modularization methods on biological networks and ranked them based on their success in the identification of closely connected disease-relevant modules. The top three performing methods can be accessed through the MONET toolbox (Tomasoni *et al.* 2020), which till now has been available as a container, but not as a webtool.

Here, we describe the Network-Oriented multi-Omics Data Analysis and Integration (NOODAI) webtool, an online platform for the combined analysis of multiple omics profiles. The tool takes as input user-provided lists of hits for different omics layers and maps them onto a high-confidence molecular interaction network. Through a user-friendly interface, NOODAI provides easy access to the top-performing modularization methods included in the MONET tool, as well as selected network centrality metrics and pathway enrichment analysis on the identified modules. Additionally, it produces several publication-ready illustrations and a detailed report in which the top central features supported by independent input datasets are functionally described.

## 2 NOODAI analysis pipeline overview

NOODAI (https://omics-oracle.com/) is a cloud-based web service that supports joint interpretation of features identified as significant in the analysis of single omics datasets. The required inputs are lists of protein or small molecule identifiers (IDs), which should be provided individually for every omics layer (genes and transcripts have to be mapped to representative UniProt IDs and ChEBI IDs should be used for metabolites). The input lists should contain elements that characterize the samples in a studied condition, such as significantly up- and down-regulated entities. In addition, optional data such as an expression change (for instance logFC) or an analogous value can be included together with the biological entity. In this case, there is a possibility to identify highly weighted network regions via network propagation. NOODAI is not constrained by the number of omics profiles and studied conditions it can accept and it is effective as a single-omics tool as well. The formatting requirements are described in the “Documentation” section of the platform and in the Supplementary Materials, available as supplementary data at *Bioinformatics* online. NOODAI maps the provided elements onto biological networks, identifies elements that are central for network connectivity and reports highly connected network modules with the MONET tool. Additionally, for each module, it provides information on whether its elements are enriched in specific signaling pathways. This gives insight into biological processes associated with a studied condition. Finally, a summary report is generated; The report includes information on the most central elements and highlights central nodes with regulatory roles.

**Figure 1.**
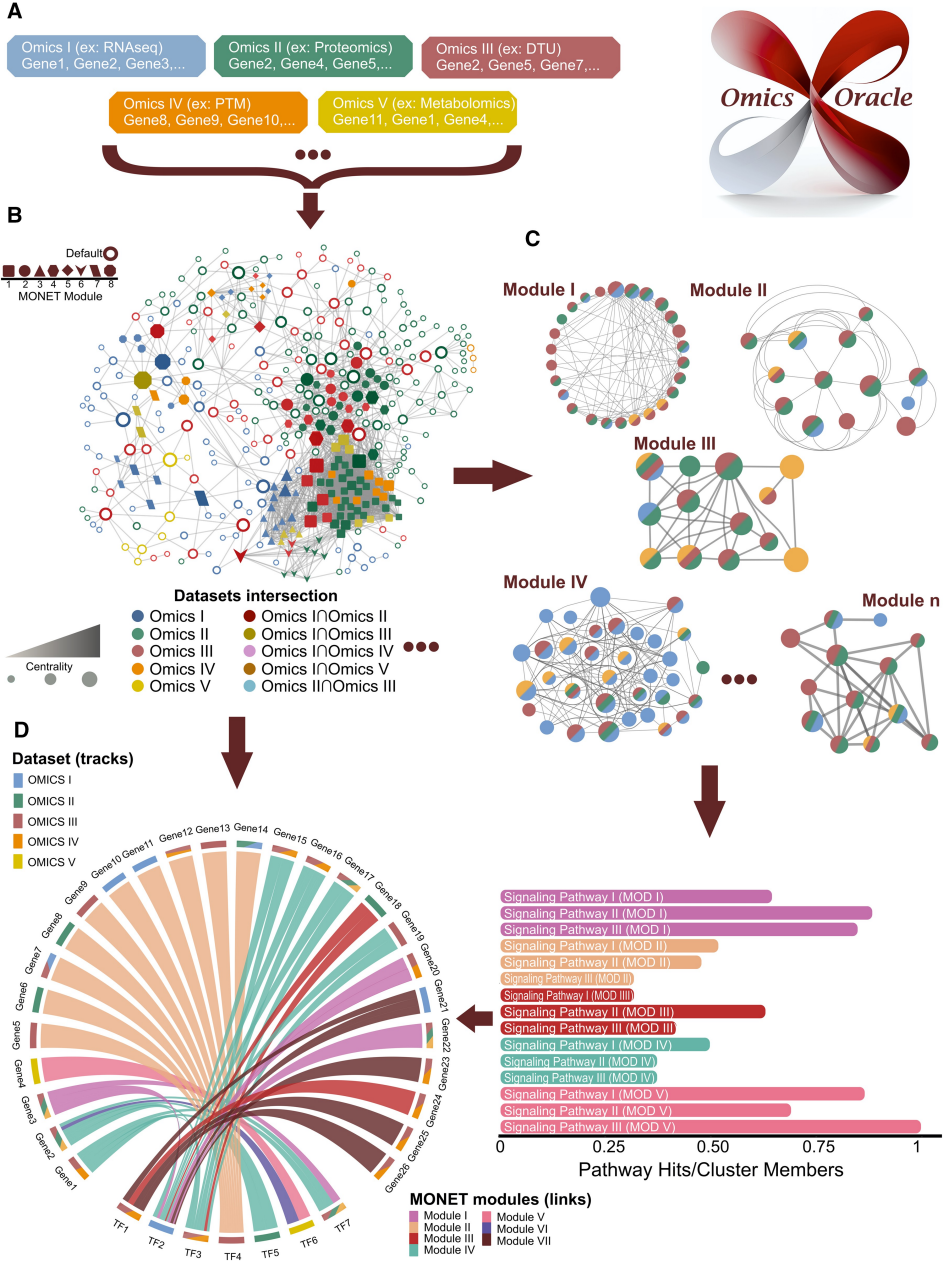
NOODAI analysis pipeline. (A) The platform takes as input lists of identifiers that are representative for each analyzed omics profile in a given condition. (B) Input lists from different omics layers are integrated into a global interaction network and a centrality score is computed for each protein in the network. (C) Following, the network is decomposed into modules using the MONET tool. (D) In addition, a summary report and illustrative plots are generated. This includes circos plots which depict connections among most central proteins and barplots that show dominant functional roles within the largest modules.

As a first step, user-provided input lists with elements relevant for individual omics layers (Fig. 1A) are mapped to a knowledge-based protein-protein interaction (PPIs) or protein-small molecule network. Pairwise interactions from STRING (Szklarczyk *et al.* 2019), BioGrid (Oughtred *et al.* 2021), and IntAct (Del Toro *et al.* 2022) databases are readily available for building these networks. They have been filtered in order to include only high-confidence interaction pairs. The NOODAI platform includes reference datasets for 13 species and it is also possible for the user to provide their own reference interactions. Interaction networks are first constructed for all individual omics layers separately and then merged through concatenation. The network with the highest number of members is used for further analysis (Fig. 1B) and smaller disconnected networks are discarded.

Following the network construction, NOODAI calculates centralities of the constituent nodes to assess the importance of individual proteins for the overall network connectivity. For this, it uses the metrics provided by the CINNA R package (Ashtiani *et al.* 2019). The default NOODAI reported metric is the Current-flow betweenness centrality. This centrality metric assesses the shortest paths that connect network elements through each respective protein, and additionally includes contributions from all possible paths by accounting for the information flow through random walks (Newman 2005). The betweenness centrality was reported to be particularly suitable for highlighting crucial elements in regulatory biological networks (Yu *et al.* 2007, Alvarez-Ponce *et al.* 2017).

Next, the global network is decomposed in order to identify key communities of highly connected elements with the MONET tool (Fig. 1C). With the default settings, the NOODAI web platform uses the modularity optimization algorithm based on the Multiresolution (M1). M1 algorithm was chosen due to the absence of a stochastic component and lower computational requirements (Arenas *et al.* 2008, Tomasoni *et al.* 2020). However, the user can also select any of the two other MONET methods, R1 or K1. As a default, unless the weighted network has been configured, an undirected and unweighted network is assumed and the desired average nodes degree in the identified modules is set to 10. If the user chooses to apply the weighted network analysis, the user provided expression changes or other numerical attributes are used as inputs for calculating edge weights based on the NetWalk approach (Komurov *et al.* 2010) followed by the weighted implementation of MONET. After the modules are defined, we assess whether proteins in the same module are enriched in any specific pathways or functional roles. A default analysis is performed using pathway annotations from the Reactome database (Milacic *et al.* 2024), but the platform also supports the use of several other databases with functional and pathway annotations. Pathway over-representation is assessed with the Fisher’s exact test and Benjamini-Hochberg’s correction at the level of modules. Regardless of whether the statistical significance threshold was reached, the three most significant pathways from the five largest modules are visualized as barcharts (Fig. 1D) and included in the NOODAI summary output. MONET configuration parameters and pathway databases that are readily available for the enrichment analysis are described in Supplementary Materials, available as supplementary data at *Bioinformatics* online and in the “Documentation” section of the platform.

Given the central role of transcription factors (TFs) in regulating cellular processes, defining phenotypes, and guiding development, NOODAI highlights key TFs in the network. The most central TFs are depicted on a circular diagram illustration together with their interaction partners, which themselves had top ranked centrality scores. This plot is included in the NOODAI summary output (Fig. 1D). The color scheme in the circular illustration entails information on signaling pathways enriched in the modules to which the shown TFs belong.

The web platform includes a demo dataset that allows users to easily understand the data formatting requirements and explore the default settings. Further information about the demo dataset can be found in the “Documentation” section of the platform, as well as in the original publication of the datasets (Totu *et al.* 2025).

Finally, to ensure a comprehensive overview of the main findings, a report including information on all central network entities (top 10%) and results of the network decomposition analysis is automatically generated. The selected nodes are listed together with basic functional description. When the central node is a known cellular regulator and functions as a TF or a kinase, or when the node is a member of a module enriched in specific signaling pathways, this information is also provided.

A comprehensive summary and performance evaluation between previous tools and the NOODAI platform are detailed in Supplementary Materials, available as supplementary data at *Bioinformatics* online, while the most important metrics are highlighted in Table 1. NOODAI requires more computational resources and time, but it is currently the only platform that provides access to the MONET tool, network analysis based on node “weights,” publication-ready plots and biologically oriented reports on main trends. Comprehensive runtime tests across datasets containing 150–1000 features showed execution times ranging from 2 to 7 min for a single comparison (see Supplementary Materials, available as supplementary data at *Bioinformatics* online).

**Table 1.** Selected metrics for comparing current multi-omics analysis tools.

	NOODAI	OmicsNet	3Omics	PaintOmics4	GeneTrail2	MergeOmics2	OmicsAnalyst
Multi-omics method	Knowledge based	Knowledge based	Data driven	Knowledge based	Knowledge based	Data driven	Data driven
Run-time for one dataset	7 min	Interactive	45 s	134 s	110 s	81 s	Interactive
Methods and report focused on biological networks	Yes	No	No	No	No	Yes	Yes
Weighted networks	Yes	No	No	No	No	No	No

## 3 Conclusions

NOODAI provides easy access to a network-based integrative characterization of significant features obtained from individual multi-omics analyses. The method maps omics hits onto knowledge-based high-confidence interaction networks, which are then analyzed both at the level of individual elements as well as at the level of tightly linked network neighborhoods or modules. NOODAI reports centrality and functional roles of individual elements and in parallel offers access to biologically relevant network decomposition methods through MONET, both for weighted and unweighted networks. The identified modules are further investigated for the enrichment in specific signaling pathways or functional roles. Human, mouse, rat and ten other species have all the required databases pre-loaded on the platform. The platform, accessible at https://omics-oracle.com, delivers main results as raw data in a tabular format as well as in a comprehensive summary report and publication-ready summary-level plots. By its design, NOODAI supports the delivery of clinically relevant insights from the combined analysis of multiple omics profiles, without the need for user’s expertise in programming and statistics.

## Supplementary Material

btaf553_Supplementary_Data

## Data Availability

No new data were generated or analysed in support of this research. We used the date from Totu *et al.* (2025) to demonstrated the webplatform usability.
