# Supplementary material for: NOODAI: a webserver for network-oriented multi-omics data analysis and integration pipeline
Source: Bioinformatics. 2025 Oct 6;41(11):btaf553. doi: 10.1093/bioinformatics/btaf553 (PMC12582364; doi:10.1093/bioinformatics/btaf553)
Supplement: btaf553_Supplementary_Data [file btaf553_supplementary_data.pdf]

NOODAI: A webserver for network-oriented multi-omics data analysis and integration pipeline

Tiberiu Totu[1,2,3], Rafael Riudavets Puig[1,2], Lukas Jonathan Häuser[1,2,3], Mattia Tomasoni[4,5], Hella Anna Bolck[6,7], Marija Buljan[1,2]

[1] Nanomaterials in Health Laboratory, Swiss Federal Laboratories for Materials Science and Technology (Empa), St. Gallen, Switzerland

[2] Swiss Institute of Bioinformatics (SIB), Lausanne, Switzerland

[3] Department of Health Sciences and Technology, Eidgenössische Technische Hochschule Zürich (ETH), Zurich, Switzerland

[4] Department of Ophthalmology, University of Lausanne, Fondation Asile des Aveugles, Jules Gonin Eye Hospital, Lausanne, Switzerland

[5] Platform for Research in Ocular Imaging, Fondation Asile des Aveugles, Jules Gonin Eye Hospital, Lausanne, Switzerland

[6] Department of Pathology and Molecular Pathology, University of Zurich and University Hospital Zurich, Zürich, Switzerland

[7] Centre for AI, School of Engineering, Zurich University of Applied Sciences (ZHAW), Winterthur, Switzerland

This supplementary material provides a detailed workflow of the NOODAI pipeline, description of the main webtool interface functionalities as well as technical validations for the default metrics.

Contents

I. NOODAI analysis pipeline
II. Demo dataset and rationale behind default settings
III. NOODAI workflow
IV. Runtime Measurements
V. Comparison with other online platforms

1. NOODAI analysis pipeline

The NOODAI software platform supports inference of the most important molecular features from the joint analysis of multiple omics layers. Its architecture relies on the usage of pre-existing interaction networks for the mapping of entities obtained through the pre-analysis of individual omics profiles. The input for the pipeline is represented by lists of features (i.e. proteins, genes, transcripts, small molecules) obtained from the analysis of one or more omics datasets, such as entities that are significantly up- or down-regulated in a specific phenotype or condition of interest. The set of features from one omics layer that characterizes a particular condition is referred to as a profile. The default configuration available on the web platform requires Uniprot protein identifiers (IDs) for the input features (i.e. representative protein IDs for differentially expressed genes) or small molecule ChEBI IDs. The analysis pipeline can be extended to accommodate any other feature lists important for characterizing the condition or phenotype of interest. For a consistent analysis, it is crucial that the features derived from various omics datasets can be logically integrated into a cohesive interaction network. The NOODAI webtool output delivers a comprehensive characterization of the studied biological conditions, presented through summary plots, tables, and a detailed report. The key elements involve analyzing the constructed networks using centrality metrics and functional characterization of network modules through the application of the MONET network decomposition tool. The latter allows for the inference of parallel signaling routes that affect molecular entities in different omics layers.

1.1. Protein-protein interaction network construction

After providing the pre-selected features as UniProt or ChEBI IDs for each of the analyzed omics layers, NOODAI maps individually features from each omics profile to the pre-existing network. When using the default settings, a protein-protein interaction (PPI) network and a protein-small-molecule network are constructed. For this, high-confidence interaction pairs collected from public knowledge databases, which are readily available on the webserver, are queried and only those for which both elements are in the input feature list are kept. The interactions are collected from STRING (Szklarczyk, et al., 2019), BioGrid (Oughtred, et al., 2021) and IntAct (Del Toro, et al., 2022) databases. STRING, database version 11.5 was filtered to include only entries with a combined score above 0.7 (starting from the complete interactions data from all sources). From the BioGRID database version 4.4.218, a mitab data file was used and only physical interactions supported by independent validations and an associated confidence value were kept. For the high confidence interactions from IntACT, the default psimitab database from 13/07/2022 was filtered to keep only interactions with a confidence score above 0.7. The following thirteen NCBI organisms' taxonomies have pre-loaded reference interactions and database files on the server and can be directly queried with protein and small molecule entries: 9913, 6239, 9615, 7955, 44689, 7227, 9031, 10090, 10116, 4932, 4896, 8364, 9606. For other organisms, custom reference interaction files must be uploaded to the webtool by the user (see Section III).

After generating the networks for each omics profile, these are merged together through concatenation. All nodes and edges in the joint network are unique. The networks are undirected and can be either weighted or unweighted (depending on the user's preference). Reliance on known interactions often results in many smaller isolated networks built from a handful of elements. In NOODAI, only the network that includes the highest fraction of the provided entities is further analyzed.

1.2. Node's centralities

After the construction of the PPI network, centrality scores are calculated for each node in the network. For this, we use the CINNA R package (Ashtiani, et al., 2019). The package computes 49 distinct centrality metrics and NOODAI reports 35 of them (some metrics demand excessive computational resources when scaling). When interpreting results for the PPI networks, which rely on the overlap of results from multi-omics analyses with known interactions, it is important to bear in mind several possible confounding trends.

Firstly, public knowledge PPI databases on one hand, miss important interactions that are not yet validated and on the other hand, tend to have well-characterized proteins over-represented due to the literature and study bias. Secondly, most network analysis methods from graph theory were not designed to convey information with biological meaning. NOODAI reports current-flow betweenness score as a default centrality measure. This metric is computed in addition to the others found in the CINNA package. Previous work has shown that betweenness centrality performed better than degree of direct interactions (i.e. "hub-ness") in identifying central regulatory genes with significant biological relevance (Alvarez-Ponce, et al., 2017; Yu, et al., 2007). Current-flow betweenness accounts for additional routes of information flow in the networks. In section II, a comparative study with the other centrality metrics is provided. By default, NOODAI determines the importance of a node based on both its centrality score and the number of omics studies supporting its presence. Nodes that are found in at least half of the input lists and which are in the top 10% of most central nodes are deemed as likely important for the studied conditions or sample groups. For these nodes, we provide functional description and specifically highlight cellular regulators, such as transcription factors and kinases.

1.3. Network decomposition

Network decomposition of the final PPI network is performed with the MONET decomposition tool (Tomasoni, et al., 2020). MOENT supports usage of algorithms represented by the M1, R1 or K1 decomposition methods. The M1 method was chosen as the default option in NOODAI due to its scalability and reproducibility (Tomasoni, et al., 2020). When the gene and/or protein expression levels are not provided, the edges are assumed to be undirected and the average desired node degree for the modules is set to 10. The MONET methods were evaluated specifically in the context of biological systems and represent the top-performing algorithms of the 'Disease Module Identification DREAM Challenge', a community effort to develop unsupervised network modularization algorithms for biological networks (Choobdar, et al., 2019). Network decomposition is applied to the joint PPI network and the identified modules of highly connected proteins will often contain members from multiple input omics layers. Proteins in the same module are expected to often be involved in the same biological processes and signaling pathways. In order to investigate which signaling pathways are associated with individual modules, annotations from the Reactome (Milacic, et al., 2024), Wikipathways (Agrawal, et al., 2024), BioCarta (Nishimura, 2001), PID (Schaefer, et al., 2009), NetPath (Kandasamy, et al., 2010), HumanCyc (Romero, et al., 2005), INOH (Yamamoto, et al., 2011) and SMPDB (Jewison, et al., 2014) signaling pathways databases are used and enrichment compared to the background of all proteins found in the full-scale PPI network is assessed. This analysis is conducted only on modules with 10 or more members. A module is assumed to be relevant for mapping independent functional routes that characterize the studied phenotype (or condition) if at least 50% of its members are associated with a specific signaling

pathway. This is particularly of interest when it helps to identify proteins that come from different omics profiles but are grouped together in the network and have shared functional relationships. Criteria for the required fraction of proteins in a module with a shared function can be adapted by the users. Additionally, the Benjamini-Hochberg (Benjamini and Hochberg, 1995) corrected p-values (FDRs) calculated for the statistical enrichment compared to the overall network are available and can be used as an additional filtering threshold. FDR thresholds are not applied by default. All pathways identified as shared among the majority of modules' members are reported to allow for a comprehensive evaluation of the roles that the module can be associated with. However, for the final presentation of results, it is advisable to filter out pathways whose members and functions strongly overlap. Cumulative interpretation of representative functions performed by individual modules can be used to generate an overview into the main signaling flow axes that underlie the studied phenotype.

1.4. Summary plots and report generation

Next, results generated in the analyses above are aggregated into summary-level plots and a comprehensive report. In addition, the edges in each of the MONET modules are extracted to support the visualization of modules using Cytoscape (Shannon, et al., 2003). An R script is provided for this and can be downloaded from the associated Zenodo repository (10.5281/zenodo.16416600). In the report provided to the user, the top three signaling pathways (based on the FDR values) for the largest five modules are illustrated in the form of a barchart where the ratio between the number of pathway members and the total number of members in the respective module is plotted. It is important to note that these top 3 pathways can be highly overlapping and have almost identical members. The overlap between the pathways can be evaluated by assessing the composition of their members. This information is available in the Excel tables in the results folder. In addition, the report includes a circular representation diagram that highlights the most central transcription factors (TF) and their connecting proteins, which themselves have a high centrality score. The connection between each interacting protein and TF is colored based on the module in which the interacting protein is found. Only interacting proteins that are among the top 30% of central nodes in the full-size networks are considered during the generation of the plot, the maximum number of represented TFs is set to 7 and the maximum possible number of their interacting proteins to 25. The barplot with pathways associated with each module and circular diagrams aim to offer the user a fast and comprehensive insight into the analyzed profiles. Besides this overview, the main results are presented in an automatically generated report in which the most central nodes are presented, and possible major signaling axes are highlighted. Information is also provided on whether the same biological entity was present in multiple input lists. The report emphasizes nodes ranked among the top 10% most central ones that are also found in at least 50% of the input omics lists. Kinases and transcription factors found within the top 15% of central nodes are also reported for the analyzed profiles. Pathways with which 70% or more of the module members were annotated are also prominently highlighted. The summary report contains information for all the characterized phenotypes (or conditions) with the circular and signaling pathways plots produced separately for each condition.

1.5. Weighted network configuration

NOODAI has implemented functionalities for analyzing weighted networks starting from nodes weights provided as positive non-negative values for each node in a column named 'Weight'. Users can input either gene (or protein) expression fold changes as metrics of their nodes, or

other numerical values that they may deem fit and descriptive. There are many approaches for finding modular structures and amplifying signals in biological networks (Mitra, et al., 2013). Based on the assumption that genes or proteins underlying similarity in phenotypes have a higher probability of interacting, one class of state-of-the-art methods are network propagation-based approaches (Cowen, et al., 2017). Specifically, to identify active modules in biological networks, network-propagation algorithms utilizing diffusion-flow and random walks (Mitra, et al., 2013). We implemented the NetWalk algorithm (Komurov, et al., 2010), which was demonstrated to be capable of identifying meaningful subnetworks in biological networks (Mitra, et al., 2013; Newaz and Milenković, 2020)

NetWalk performs network propagation by random walks, which are biased towards nodes with high weights (as indicated, e.g., by fold changes) using a restart mechanism (Komurov, et al., 2010). The method of random walks has been well established for structural analyses of networks. Their application to graphs has long been studied (Lovász, et al., 2021), and they have been successfully used on complex networks (Rosvall and Bergstrom, 2008). NetWalk works using a random walk on an interaction graph, where transition probabilities represented by a propagation matrix P are determined by the data values w (e.g., fold changes) and the adjacency matrix of the network A with a restart probability q at any step of the walk provided by the user as a parameter. The transition probabilities (without restart) P are the normalized data values as $P_{ij} = w_j/(Aw)_i$. To incorporate the restart mechanism, the restart propagation operator Q is defined as $Q_{ij}=w_i/\|w\|$. The final combined propagation operator is then given as a combination of P and Q by $S=(1-q)P+qQ$. NetWalk calculates the output node weights as the stationary state of the stochastic process described by the propagation operator S. By the Perron-Frobenius theorem, this stationary state is given by the eigenvector g of S with the largest eigenvalue of $\lambda=1$. The weighted adjacency matrix W is then calculated as the edge flux $W_{ij}=g_iP_{ij}$.

If some of the omics layers do not have a weight column, a uniform weight of 1 is assigned to each edge. In order to aggregate the edge weight from multiple omics layers, the final weights for edges are summed together across different omics layers and scaled between 0 and 1.

2. Demo dataset and rationale behind default settings

Macrophages are immune cells that exhibit a high degree of phenotypic plasticity and are known to be involved in a number of pathological conditions (Totu, et al., 2024). In the context of cancer, macrophages are double-edge swords exhibiting both pro- and anti-tumorigenic phenotypes by either supporting or inhibiting anti-tumor inflammatory activity. Even though extensively studied, our current understanding of the main phenotypic drivers is largely confined to pro-inflammatory macrophages. Understanding the differences in the main signaling routes and phenotypic traits between different types of macrophages could impact the rational design of novel cancer therapeutics.

Here, we applied the NOODAI pipeline on previously generated omics datasets for human blood-derived pro-inflammatory *in vitro* M1 (LPS and IFN-γ stimulated) and *in vitro* immunosuppressive M2a (IL-4 and IL-13 stimulated) and M2c (IL-10)-macrophages (Totu, et al., 2024). The datasets included next-generation sequencing transcriptome measurements that indicated gene expression levels and allowed inference of splice isoforms as well as mass spectrometry-based proteomics and phosphoproteomics quantitative measurements. These datasets are available in a reduced form as Demo on the web platform. Due to resource management, the Demo dataset contains only 20% of the original omics data, but nonetheless preserves the original trends (Totu, et al., 2024). Throughout this section, we will follow each analysis step with direct application to the Demo data and present the rationale behind the default settings chosen for the NOODAI pipeline.

The analyzed omics profiles in M1, M2a and M2c macrophage states were represented by features (proteins and genes) that were found to be upregulated in one phenotype when directly compared to another. The criteria for this was a log2FC threshold of 1 and FDR threshold of 0.05 for (phospho-)proteomics and a log2FC 2 and FDR threshold 0.05 for transcriptomic data. Pairwise comparisons between M1 and each of the M2 states were performed on all available omics layers and significantly up- and down-regulated entities were noted separately. This resulted in the six input lists in the demo datasets for each analyzed layer, with the exception of the splicing layer which included only differentially used transcripts.

For each input list, i.e. significantly up or down-regulated entities, PPI networks are built for each omics layer and then a joint network is generated through concatenation. Next, current-flow betweenness centrality is calculated for each node within the networks. In case of the demo dataset, this highlighted proteins such as STAT1, NFKB2 and PML for pro-inflammatory M1 macrophages, and CSF1R and ITSN1 for M2 macrophages. Central regulatory roles of these proteins in the respective phenotypes are supported by numerous previous studies (Chen, et al., 2023; Ordentlich, 2021).

In order to assess in more detail the role of the nodes ranked in the top 10% of the current-flow betweenness centrality scores in underlying macrophages clinical phenotypes, we performed analysis with the VOSviewer bibliometric tool (Van Eck and Waltman, 2014). For this, all clinical studies on macrophages from Embase from 2012 onwards were screened using the search term: "((human OR primary) NEXT/3 macrophage*) AND [humans]/lim AND [clinical study]/lim)". Only the terms with at least 3 mentions were selected. This showed that at least one tenth of the most central nodes in each contrast was mentioned in the clinical studies that involved macrophages. To evaluate NOODAI-computed centrality algorithms, the percentage of network proteins associated with clinical trials that fall within the top 10% of central nodes, relative to the total number of clinically associated network proteins, was used as a benchmark.

On average, current-flow betweenness centrality aggregates 30% of the network proteins associated with clinical trials within the top 10% of central nodes (Figure S1). Eccentricity, Group and Topological Coefficient centralities show similar performance.

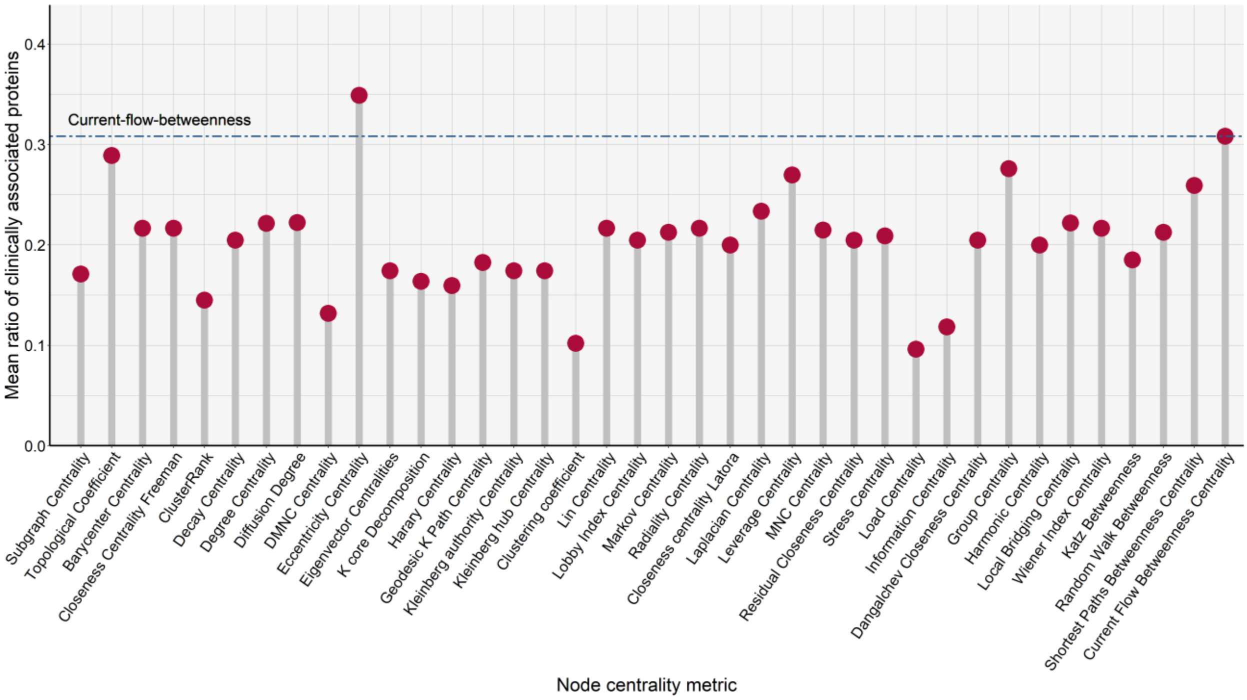

Figure S1. The average ratio of clinically associated proteins within the top 10% of central nodes, relative to the total number of clinical proteins found in the datasets, for each of the centrality metrics computed by NOODAI.

Following, the MONET network modularity optimization method (M1) is used for the network decomposition. For the resulting modules, we assess whether the majority of their members are associated with specific signaling routes. For M1 macrophages, the main parallel signaling routes included IFN-γ, Toll-like receptor signaling, NF-κB signaling, NOD-like receptor signaling, TNF and JAK-STAT signaling. This convincingly recapitulated the known biology of pro-inflammatory macrophages (Chen, et al., 2023). The MONET modules associated with immunosuppressive M2 *in vitro* macrophages were found to be linked with cell-adhesion-related pathways, phagocytic signaling routes, and the GTPase cycle, processes previously linked to this state. This highlighted the capability of the MONET decomposition tool in extracting subnetworks associated with the most important signaling routes in different macrophage phenotypes.

3. NOODAI workflow

The webtool, hosted at omics-oracle.com, features a comprehensive user manual that explains input parameters and provides guidance on general usage. The platform relies on DigitalOcean cloud service infrastructure and incorporates essential security configurations to ensure the protection of the uploaded data. A cloud solution was chosen as it is easily scalable. The interface was designed in R Shiny (Chang, et al., 2021) and it makes use of *uuid* (Urbanek and Ts'o, 2022), *shinyjs* (Attali, 2021), *bslib* (Sievert, et al., 2023) and *shinyWidgets* packages (Perrier, et al., 2023) as well as *parallel* (R Core Team, 2022) and *callr* (Csárdi and Chang, 2022) that make possible the dynamic use of the web service. For each client submission, a background process is started which allows users to close their browsers while they wait for the results. The omics-oracle interfaces and user manuals were designed in HTML and CSS with bootstrap v5. The source code of the NOODAI pipeline is available on GitHub (Totu, 2024). NOODAI relies on *ggplot2* (Wickham, 2020) for graphical design and *biomaRt* (Durinck, et al., 2009) for mapping UniProt IDs to NCBI IDs when species other than those for which the pre-loaded datasets are available are analyzed. Additional packages that are used during the analysis are *devtools* (Wickham, et al., 2022), *reshape* (Wickham, 2007), *moments* (Komsta and Novomestky, 2022), *EnvStats* (Millard, 2013), *readr* (Wickham, et al., 2023), *readxl* (Wickham, et al., 2023), *openxlsx* (Schauberger and Walker, 2023), *tidygraph* (Pedersen, 2023), *ggpubr* (Kassambara, 2023), *gtools* (Bolker and Warnes, 2022), *stringr* (Wickham and Wickham, 2019), *ggcorrplot* (Kassambara, 2019), *centiserve* (Jalili, et al., 2015), *RColorBrewer* (Neuwirth and Brewer, 2014), *gridBase* (Murrell, 2014), *circlize* (Gu, et al., 2014), *clusterProfiler* (Wu, et al., 2021), *igraph* (Csardi and Nepusz, 2006)*, dplyr* (Wickham, et al., 2023) and *CINNA (Ashtiani, et al., 2019)*. The webtool is released under GPL license v3.0.

The NOODAI webtool workflow consists of two main parts: data upload together with the selection of input parameters and the subsequent results download. To access the results after the analyses are finished, the user navigates to the "Results download" tab. Here, the files can be downloaded as a compressed archive using the assigned token ID generated upon submission. The results of an analysis are stored on the server for a maximum of 5 days, and the associated token IDs are randomly generated to ensure data security.

Each of the 4 sections of the analysis pipeline can be considered as an independent Segment of the workflow that can be individually accessed through the platform (Network construction and node centrality – Network decomposition (MONET) – Module signaling pathways analysis – Summary plots and report generation). The main interface (Figure S2A) allows users to efficiently run the entire analysis pipeline (all 4 Segments) requiring only a few inputs. Each field has an associated description. Most fields have default values, while some require mandatory input from the user. The fields marked in Figure S2A are described as follows:

1. In the "Omics data archive" field users are required to upload a single archive that contains one Excel file for each omics layer. Each Excel file must include at least one sheet. The names of each sheet should match precisely the comparison groups provided in the "Conditions comparison (contrasts)" field. In the case of the demo dataset, an Excel file for each omics layer contains 6 sheets with the following names “M1vsM2a”, ”M2avsM1”, “M1vsM2c”, ”M2cvsM1”, “M2avsM2c” and “M2cvsM2a”. The Excel sheets must include a column named “UniProt_ChEBI”. This column should have only UniProt or ChEBI IDs.

2. Under "Conditions comparison (contrasts)", users are required to upload the comparison groups. In the case of the demo dataset, a comparison group is represented by the elements upregulated in one phenotype/condition compared to another, and the input for this field has the following structure:

   “M1vsM2a,M2avsM1,M1vsM2c,M2cvsM1,M2avsM2c,M2cvsM2a”. Each contrast is composed of Condition Names joined by “vs” and must be identical to the names in the Excel sheets in the uploaded omics files. Different contrasts must be separated by commas.

3. The "Use demo data" button allows automatic upload of the demo dataset available on the platform with pre-configured input parameters. Pressing the Submit button starts the analysis of the demo dataset.

4. The "Download demo data" button allows the users to download precompiled results files that are generated when running the default analysis pipeline for the Demo dataset.

5. NOODAI can compute the centralities metrics and apply MONET decomposition on weighted networks. For this, edge weights are computed from input node weights using the NetWalk algorithm. The "Weight penalty" parameter on the platform sets the NetWalk restart probability.

6. The default “MONET method” parameters are for the MONET M1 method. Users have the option to change the method to R1 or K1. The avgk parameter that influences the average module size can also be adjusted as needed. The format must follow the one in the default setting.

7. The "Draw networks" option can be activated in order to generate the Cytoskape networks associated with the identified MONET modules.

8. A user can choose to use pre-formatted interaction files available on the server or add a new PPI dataset by clicking on the "Add custom databases" button. To use the webtool for organisms other than the 13 for which the interaction datasets are pre-loaded or to use other types of interactions, it is mandatory to upload a custom pre-formatted interaction file.

9. Pathway over-representation analysis computed on the modules can be performed with multiple signaling pathway databases, including Reactome, Wikipathways, BioCarta, PID, NetPath, HumanCyc, INOH, and SMPDB. Users are responsible for complying with the licensing terms of these databases.

10. The default "BioMart dataset" field is pre-set for mapping human protein IDs, but other species can be selected from the list. The IDs are mapped with the biomaRt R package.

11. The optional “Email address” field allows users to receive an email notification when the analysis is completed.

When the “Submit” button is pressed, the analysis starts and a unique token is generated. This token should be used in the “Results download” tab for retrieving the final results when the analysis is finished. Alternatively, the provided web link can be used for the download.

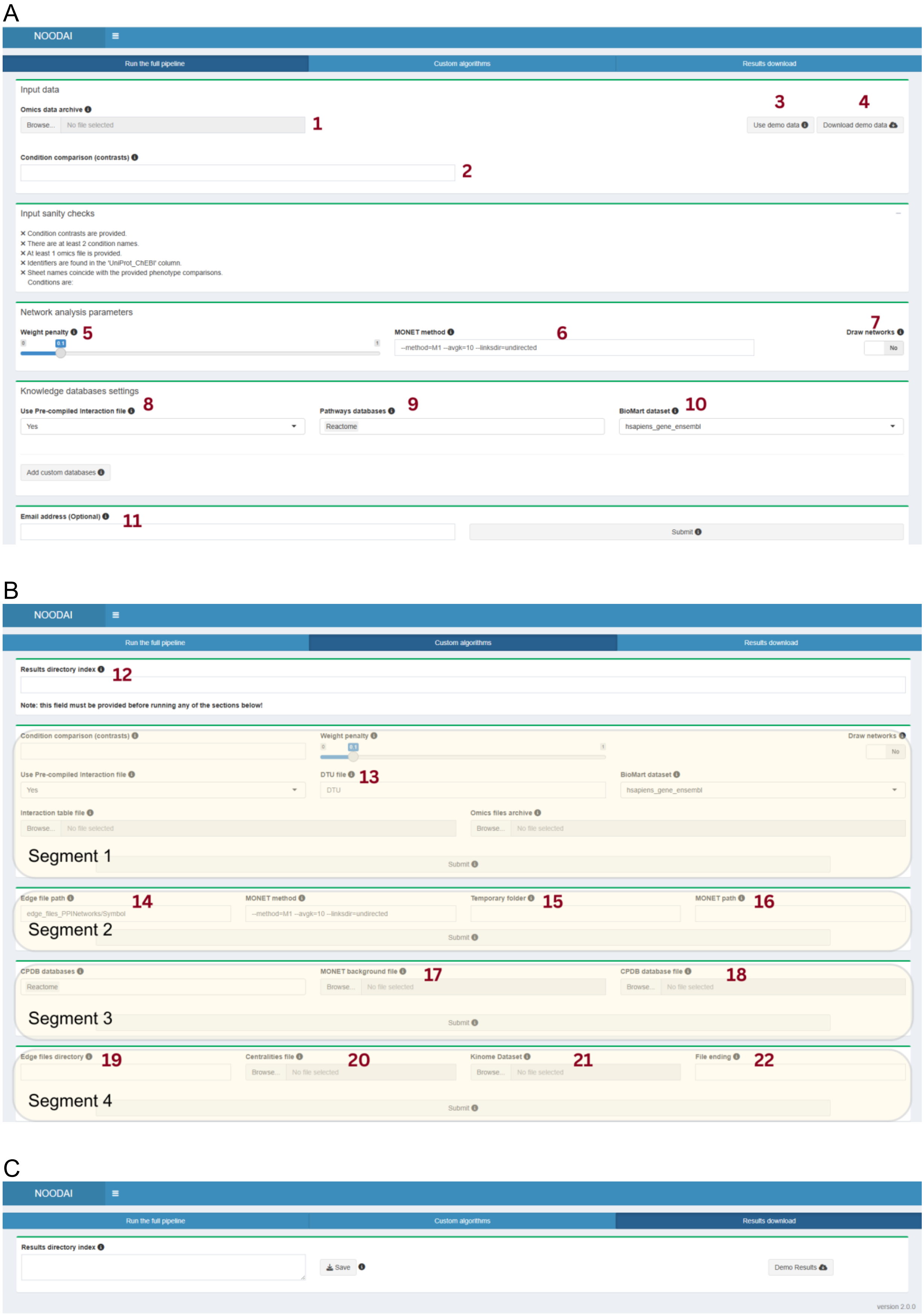

Figure S2. NOODAI interface. Fields are assigned numbers based on the order of appearance: A) Complete pipeline run B) Individual algorithms tab and C) Results download panel.

In addition to running the entire analysis pipeline in a single run, the NOODAI interface allows users to customize the parameters of each individual analysis segment (Figure S2B) and run the segment separately. This can be done after the complete analysis pipeline is finalized from the “Run the full pipeline” tab and a token is assigned to it. The following description applies to the fields within the “Custom algorithms” tab that support optimization of parameters for individual segments:

12. An analysis token generated for the execution of the full analysis pipeline can be used in the "Results directory index" for running an analysis segment independently.
13. If the input omics datasets include splicing data please enter the exact Excel file name (without extension) into the "DTU file" field.
14. By default, the names reported for the nodes in the MONET modules and in the results of the pathway analysis are official gene names. They are stored as Gene Names in the "Symbol" folder on the server. To use UniProt IDs instead of Gene names, users can use the "Edge file path" field and change there the folder path from “Symbol” to “Uniprot”.
15. - 16.These two fields are relevant when the pipeline is downloaded from GitHub and used locally. The webtool was designed to be run locally as well. For this, it is necessary to create a temporary folder in which intermediate files and results from the MONET analysis are stored. The name of the folder should be defined in the “Temporary folder” field. The temporary files created in this folder are automatically deleted at the end of the data processing. In addition, when NOODAI is run locally, the MONET executable path must be provided in the “MONET path” field.17. There is also a possibility for the users to use a different background list for the overrepresentation (i.e. enrichment) analysis (default are all entities from the joint PPI network). An alternative background file can be uploaded using the "MONET background file" field.
18. The pathways knowledge-based datasets used for the over-representation analysis are extracted from the CPDB database. The user can change and upload their own pathway annotations by using the “CPDB database file” field and following the same formatting scheme.
19. By default, circular plots and the final summary report use official gene names. Users preferring to use UniProt IDs can switch to the “Uniprot” folder as mentioned in point 14 above. The "Edge files directory" field must match the "Edge file path". Therefore, both fields should be formatted identically when changing the edge folder.
20. NOODAI relies on a PPI network constructed by concatenating the networks from individual omics profiles. Results output files also include centrality metrics for individual omics layers. If the users wish to generate the summary report and plots for a specific omics input dataset, they can upload the respective centrality file under the "Centralities file" field. If one would like to have the summary report and plots for a different metric than the current-flow betweenness centrality, the file can be modified offline and uploaded again on the platform.
21. The user can choose to use a different list of kinases instead of the default one available on the server. This can be uploaded under the "Kinome Database" field. This may be also used if the user wants to replace kinases with phosphatases, epigenetic regulators or other class of proteins.

22. By default, the "File ending" field is set to "Total", indicating that the network built from all omics layers is analyzed for the final plots and report. In order to generate plots only for a certain omics dataset, another centrality file should be used, as described in point 20 above, and the “File ending” field should be updated with the name of the input omics file name.

The "Results download" tab (Figure S2C) allows users to download the results generated from the analysis of the demo dataset or to retrieve the results from their own analysis in the form of an archive file.

The platform offers users flexibility with regard to the type of data that can be analyzed and the parameters used for the analysis. Its default inputs are protein and small molecule IDs and it has pre-loaded datasets for thirteen species, but the pipeline can be used for any other species and omics features as well. For this, users must provide the correct BioMart dataset name, a pre-formatted feature-feature interaction file, a reference signaling pathway database, and a custom TF dataset. For the pre-loaded species, only the BioMart dataset name must be properly set up. For more details, the users are encouraged to consult the online documentation available on the platform.

4. Runtime Measurements

User-perceived runtime was assessed as a function of dataset size using two independent approaches. First, datasets of varying sizes were created by random subsampling from the Demo dataset (coined real-world dataset). Second, synthetic datasets were generated by random sampling from a predefined list of proteins (synthetic dataset). Dataset size is defined as the number of proteins per modality. The measured runtimes provide an estimate of the processing duration a user can expect and illustrate how runtime scales with increasing dataset size. The associated code and datasets are available in the Zenodo repository (Totu, 2024).

4.1. Runtime Measurements for Real-World Data

To assess NOODAI's runtime on real-world data across varying dataset sizes, the measured dataset was randomly subsampled to contain between 150 and 400 proteins per modality. Runtime measurements for each subsampled dataset are presented in Figure S3 as a stacked bar chart, illustrating the contribution of individual analysis steps to the total runtime. Overall runtimes ranged from 134 seconds for the smallest dataset (150 proteins per modality) to 197 seconds for the largest (400 proteins per modality).

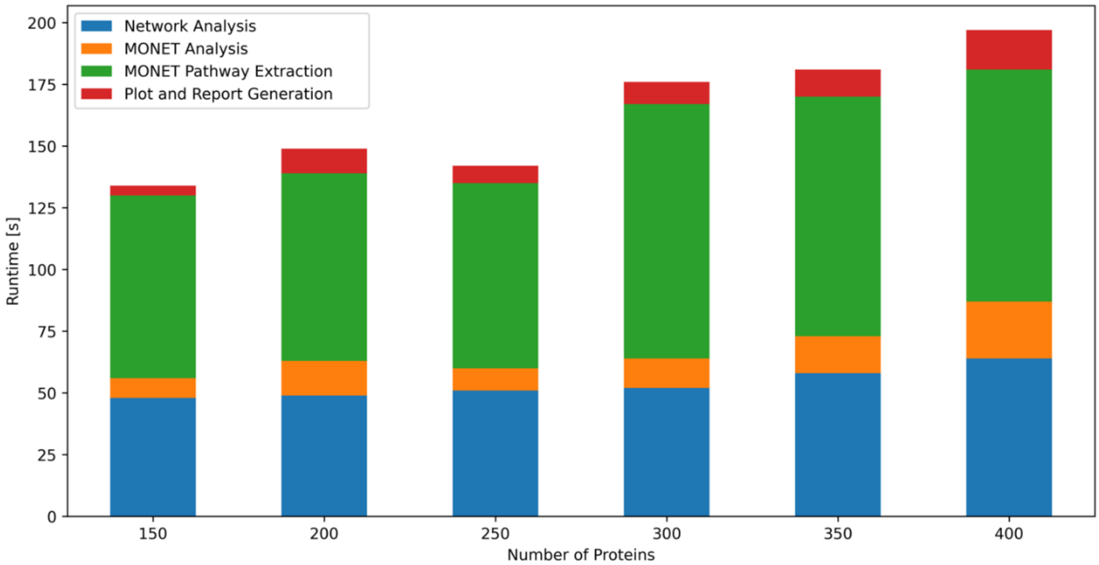

Figure S3. Total runtimes of NOODAI for subsampled data from the Demo dataset of varying sizes, defined by the number of proteins per modality.

Among the four processing steps, pathway extraction performed by MONET accounts for the largest proportion of total runtime, ranging from 48% to 59% (Figure S4). In contrast, the MONET analysis and the generation of plots and reports contribute smaller portions, ranging from 6% to 12% and 3% to 8%, respectively. Network analysis comprises 30% to 36% of the total runtime. Overall, we observe a 1.5-fold increase in total runtime corresponding to a 2.7-fold increase in dataset size.

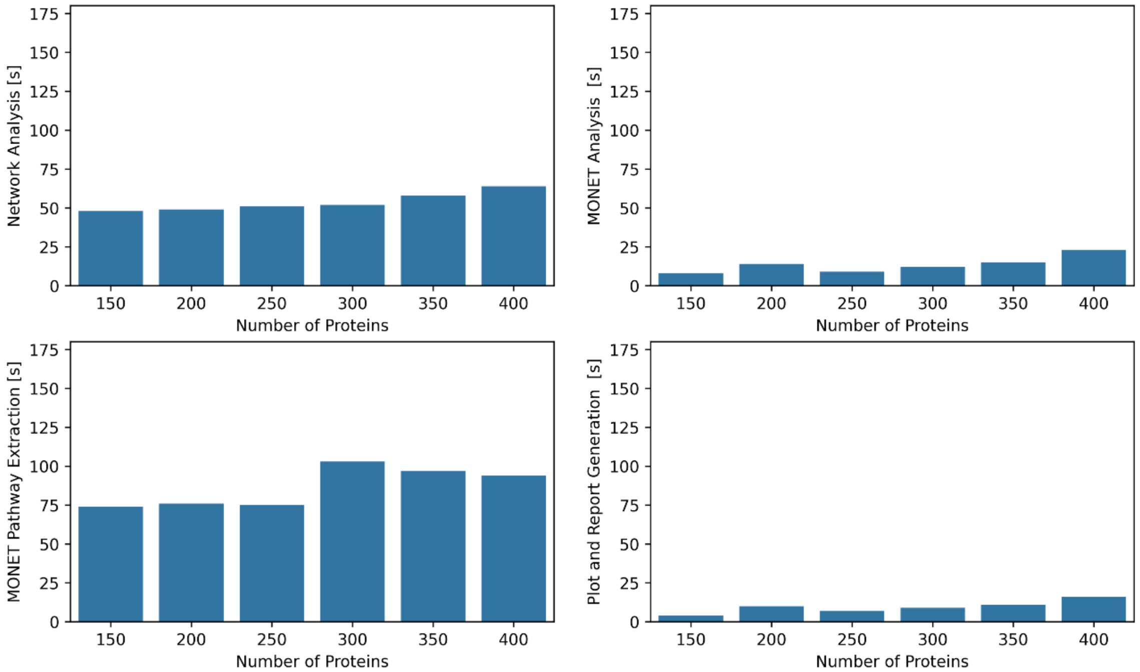

Figure S4. Runtimes of individual processing steps of NOODAI on subsampled data from the Demo dataset of varying sizes, defined by the number of proteins per modality

4.2. Runtime Measurements for Synthetic Data

To evaluate the runtime behavior of NOODAI on larger datasets, we synthesized data by randomly sampling from a list of 6000 proteins across all modalities, with sample sizes ranging from 200 to 1000 proteins per modality (Figure S5). The corresponding runtimes increase from 121 seconds for the smallest dataset (200 proteins per modality) to 442 seconds for the largest (1000 proteins per modality).

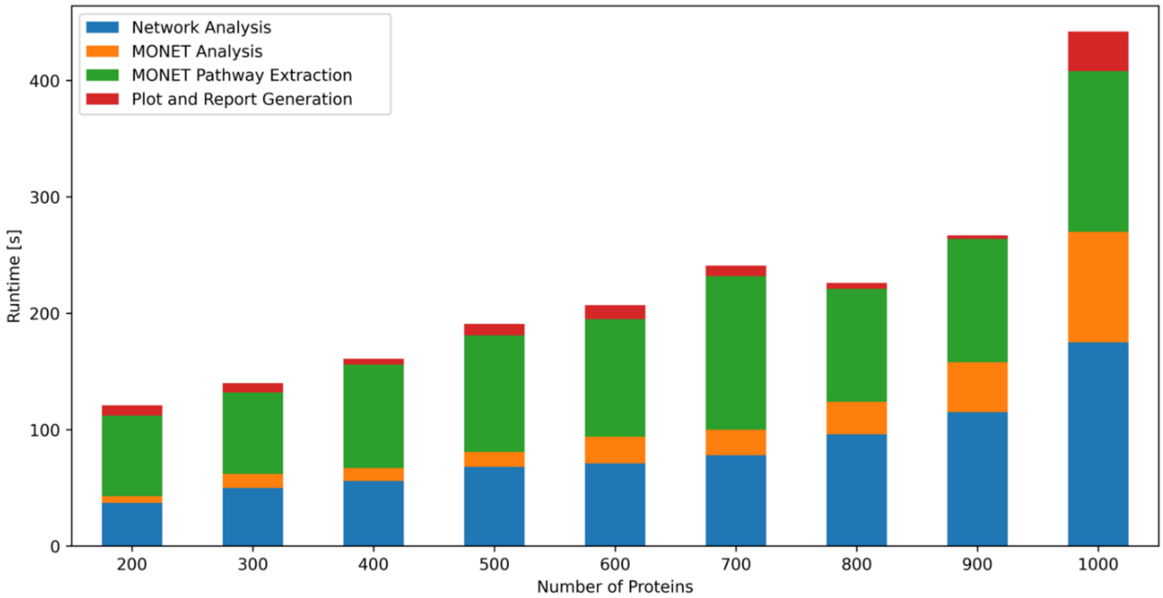

Figure S5. Total runtimes of NOODAI for synthetic datasets of varying sizes, defined by the number of proteins per modality.

Similarly to the previous dataset, the individual processing steps of NOODAI contributed unequally to total runtime (Figure S6). Among the four steps, either pathway extraction by MONET or network analysis contributed the largest share to total runtime, ranging from 31–

57% and 31–42%, respectively. For datasets with 800 or fewer proteins per modality, pathway extraction was more time-intensive, whereas network analysis dominated for datasets with 900 or more proteins. In comparison, MONET analysis and the generation of plots and reports accounted for smaller fractions of the total runtime, contributing 5–21% and 1–8%, respectively. These results suggest that network analysis part exhibits a less favorable scalability and is likely the main determinant of runtime for large datasets.

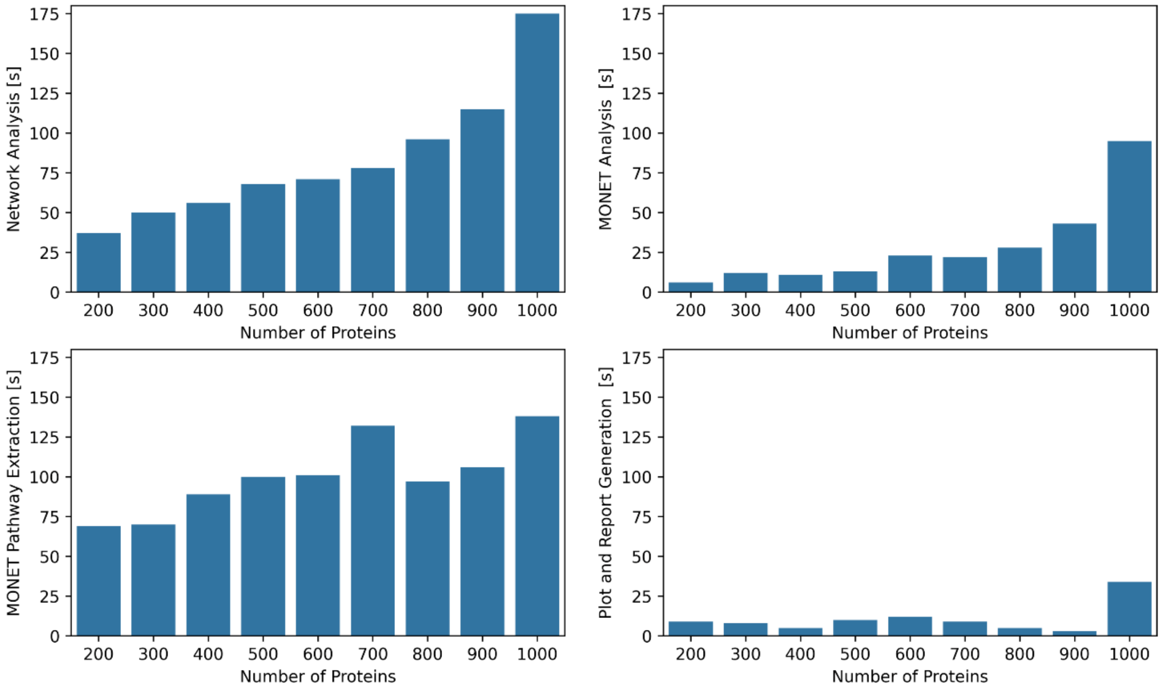

Figure S6. Runtimes of individual processing steps of NOODAI for synthetic datasets of varying sizes, defined by the number of proteins per modality.

5. Comparison with other online platforms

A number of web platforms have been developed for multi-omics analysis, implementing a wide range of knowledge-based and data-driven integration frameworks, each with distinct strengths and limitations. NOODAI stands out by employing biologically oriented network analysis methods specifically designed to analyze biological interaction networks while remaining accessible and easy to interpret. A comprehensive comparison of different web platforms is presented in Supplementary Table 1, where NOODAI is evaluated alongside state-of-the-art platforms such as OmicsNet (Zhou, et al., 2022) and OmicsAnalyst (Zhou, et al., 2021) as well as highly used platforms such as 3Omics (Kuo, et al., 2013), PaintOmics4 (Liu, et al., 2022), GeneTrail2 (Stöckel, et al., 2016) and MergeOmics2 (Ding, et al., 2021). For the performance metric testing, 100 features, a minimum of 3 omics layers and one contrast were used for the benchmark.

Supplementary Table 1: Comparison of current OMICS network analysis tools. Dots indicate the given score for the different feature classes. (weak: ● fair: ● ● strong: ● ● ●).

	NOODAI	OmicsNet	3Omics	PaintOmics4	GeneTrail2	MergeOmics2	OmicsAnalyst
General characteristics	●●●	●●	●	●●	●	●●●	●●●
Input	Excel files with pre-selected features for each omics layer in an archive	Text list of features for each omics layer, graph files	CSV files with variations under experimental conditions	TSV files of omics datasets and lists of significant features	TSV file with list of relevant entities; BED files; GSE identifier	TSV files; marker association file	CSV file with samples in rows and factors in columns
Input data check	Strong input checks	None	checks number of rows and columns, but no semantic checks	None	checks only correctness of identifiers	Strong input checks	Checks number of rows and columns, but no semantic checks
Input IDs types	UniProt, ChEBI	NCBI ID, Ensembl Gene ID, Ensemble Transcript ID, Official Gene Symbol, Refseq ID, GeneBank ID	NCBI Gene IDs, UniProtKB IDs and PubChem CIDs	NCBI IDs	UniProt, Gene Symbols, NCBI/Ensembl; GSM identifiers from GEO database	Entrez ID, Ensembl ID, HUGO symbol	NCBI ID, Ensembl Gene ID, Ensemble Transcript ID, Uniprot ID, HUGO symbol
Type of multi-omics integration	Knowledge-based	Knowledge-based	Data-driven	Knowledge-based	Knowledge-based; no integration of different omics layers	Data-driven	Data-driven
Biologically-oriented results inference methods (Biology-driven methods)	Yes, by using MONET	Graph files generated from OmicsNetR package or Cytoscape provide biologically-oriented results	No	No	No	Yes, but only in combination with pharmomics	Yes, e.g. Data Integration Analysis for Biomarker Discovery (DIABBLO)
Number of species	13 default and user defined	10	1 (only human)	70	agnostic (16 databases)	2 (human or mouse)	2 (human or mouse), or agnostic
Email notification	Yes	No	No	No	No	Yes	No
Required to keep the browser open	No	Yes	Yes	No	Yes	No	Yes
Results temporarily stored on the server	Yes	No	No	Yes	No	Yes	No
Maximum number of omics layers	Unlimited	8	3	Unlimited	4, but only individually analyzed	4	5

	NOODAI	OmicsNet	3Omics	PaintOmics4	GeneTrail2	MergeOmics2	OmicsAnalyst
Analysis characteristics	●●●	●●	●	●	●●	●●	●●●
Number of computed centrality metrics	36	5	NaN	NaN	NaN	NaN	NaN
Weighted network analysis capabilities	Yes	No	No	No	No	No	No
Network modularization method	MONET (M1/R1/K1)	InfoMap, Walktrap, Label Propagation	Just a user-provided cutoff for weight of edges	Only hub analysis for metabolites	NaN	wKDA	Not applicable
Enrichment analysis	Yes	Yes	Yes	Yes	Yes	Yes	Yes
Customized knowledge databases	Choice between 8 pathway databases, or own database file	Choice between several DBs for protein, miRNA, metabolites and TF-gene; and own graph file	No	Always uses KEGG plus two optional DBs	choice between 16 DBs	choice between 13 DBs	Choice between KEGG, Reactome, GO, Panther, and Motif
Customized analysis pipeline	Highly customizable pipelines	Highly customizable pipelines	Only three parameters to set	No, only threshold setting and feature selection	Only choice between overrepresentation and gene set enrichment analysis	Highly customizable pipelines	Highly customizable pipelines
Single analysis step repetition	Yes	Yes	No	No	No	No	Yes

	NOODAI	OmicsNet	3Omics	PaintOmics4	GeneTrail2	MergeOmics2	OmicsAnalyst
Run-time metrics	●	●●●	●●	●	●	●●	●●
Runtime for one Full dataset	7min	interactive tool	45s	134s	110s (only a single omics layer)	81s	interactive tool
Task Completion Time	8 min	5 min	1 min	4 min	2 min (only a single omics layer)	3 min	5 min
Multiple simultaneous analyses	Yes	No	Yes	No	No	No	Yes
Responsiveness	Good	Waiting time between analysis steps	Good	Good	Good	Good	Waiting time between analysis steps
Caching	No	No	No	Yes, by account login	Yes, by account login	Yes, and provides session id	No

	NOODAI	OmicsNet	3Omics	PaintOmics4	GeneTrail2	MergeOmics2	OmicsAnalyst
Results presentation	●●●	●●●	●	●●●	●●	●●●	●●●
Summary diagrams	Yes	Yes	Yes	Yes	Yes	Yes	Yes
Summary report	Yes	No	No	No	No	No	No
Online network visualization	Yes	Yes, interactive	Yes	Yes	No	Yes	Yes
Complete analysis output	Yes	Yes	Yes, but not downloadable	Yes	Yes	Yes	Yes

Bibliography

Agrawal, A., *et al.* WikiPathways 2024: next generation pathway database. *Nucleic Acids Research* 2024;52(D1):D679-D689.

Alvarez-Ponce, D., Feyertag, F. and Chakraborty, S. Position matters: network centrality considerably impacts rates of protein evolution in the human protein–protein interaction network. *Genome biology and evolution* 2017;9(6):1742-1756.

Ashtiani, M., Mirzaie, M. and Jafari, M. CINNA: an R/CRAN package to decipher Central Informative Nodes in Network Analysis. *Bioinformatics* 2019;35(8):1436-1437.

Attali, D. 2021. shinyjs: Easily Improve the User Experience of Your Shiny Apps in Seconds. Release 2.1.0

Benjamini, Y. and Hochberg, Y. Controlling the false discovery rate: a practical and powerful approach to multiple testing. *Journal of the Royal Statistical Society: Series B (Methodological)* 1995;57(1):289-300.

Bolker, B. and Warnes, G. Lumley, T. gtools: Various R programming tools. R package version 3.9. 4. In.; 2022.

Chang, W., *et al.* shiny: Web Application Framework for R. R package version 1.6. 0. 2021. *URL* *https://CRAN.R-project.org/package=shiny* 2021.

Chen, S., *et al.* Macrophages in immunoregulation and therapeutics. *Signal Transduction and Targeted Therapy* 2023;8(1):207.

Choobdar, S., *et al.* Assessment of network module identification across complex diseases. *Nature Methods* 2019;16(9):843-852.

Cowen, L., *et al.* Network propagation: a universal amplifier of genetic associations. *Nature Reviews Genetics* 2017;18(9):551-562.

Csárdi, G. and Chang, W. 2022. callr: Call R from R. Release 3.7.3

Csardi, G. and Nepusz, T. The igraph software package for complex network research. *InterJournal, complex systems* 2006;1695(5):1-9.

Del Toro, N., *et al.* The IntAct database: efficient access to fine-grained molecular interaction data. *Nucleic Acids Research* 2022;50(D1):D648-D653.

Ding, J., *et al.* Mergeomics 2.0: a web server for multi-omics data integration to elucidate disease networks and predict therapeutics. *Nucleic Acids Research* 2021;49(W1):W375-W387.

Durinck, S., *et al.* Mapping identifiers for the integration of genomic datasets with the R/Bioconductor package biomaRt. *Nature protocols* 2009;4(8):1184-1191.

Gu, Z., *et al.* " Circlize" implements and enhances circular visualization in R. *CRAN* 2014.

Jalili, M., *et al.* CentiServer: a comprehensive resource, web-based application and R package for centrality analysis. *PloS one* 2015;10(11):e0143111.

Jewison, T., *et al.* SMPDB 2.0: big improvements to the Small Molecule Pathway Database. *Nucleic Acids Research* 2014;42(Database issue):D478-484.

Kandasamy, K., *et al.* NetPath: a public resource of curated signal transduction pathways. *Genome Biology* 2010;11(1):R3.

Kassambara, A. ggcorrplot: Visualization of a Correlation Matrix using'ggplot2'. *R package version 0.1* 2019;3.

Kassambara, A. ggpubr:"ggplot2" based publication ready plots. *R package version 0.6. 0* 2023;438.

Komsta, L. and Novomestky, F. 2022. moments: Moments, Cumulants, Skewness, Kurtosis and Related Tests. Release 0.14.1

Komurov, K., White, M.A. and Ram, P.T. Use of data-biased random walks on graphs for the retrieval of context-specific networks from genomic data. *PLoS computational biology* 2010;6(8):e1000889.

Kuo, T.-C., Tian, T.-F. and Tseng, Y.J. 3Omics: a web-based systems biology tool for analysis, integration and visualization of human transcriptomic, proteomic and metabolomic data. *BMC systems biology* 2013;7:1-15.
Liu, T., *et al.* PaintOmics 4: new tools for the integrative analysis of multi-omics datasets supported by multiple pathway databases. *Nucleic Acids Research* 2022;50(W1):W551-W559.
Lovász, L., *et al.* Random walks on graphs: A survey, 1993. *Cited in* 2021:21.
Milacic, M., *et al.* The reactome pathway knowledgebase 2024. *Nucleic Acids Research* 2024;52(D1):D672-D678.
Millard, S.P. 2013. EnvStats: An R Package for Environmental Statistics. Release 2.8.0
Mitra, K., *et al.* Integrative approaches for finding modular structure in biological networks. *Nature Reviews Genetics* 2013;14(10):719-732.
Murrell, P. gridBase: Integration of base and grid graphics. *R package version 0.4-7* 2014:24.
Neuwirth, E. and Brewer, R.C. ColorBrewer palettes. *R package version* 2014;1(4).
Newaz, K. and Milenković, T. Inference of a dynamic aging-related biological subnetwork via network propagation. *IEEE/ACM transactions on computational biology and bioinformatics* 2020;19(2):974-988.
Nishimura, D. BioCarta. *Biotech Software & Internet Report: The Computer Software Journal for Scient* 2001;2(3):117-120.
Ordentlich, P. Clinical evaluation of colony-stimulating factor 1 receptor inhibitors. In, *Semin Immunol*. Elsevier; 2021. p. 101514.
Oughtred, R., *et al.* The BioGRID database: A comprehensive biomedical resource of curated protein, genetic, and chemical interactions. *Protein Science* 2021;30(1):187-200.
Pedersen, T.L. tidygraph: A Tidy API for Graph Manipulation [R Package]. In.; 2023.
Perrier, V., Meyer, F. and Granjon, D. 2023. shinyWidgets: Custom Inputs Widgets for Shiny. Release 0.7.6
R Core Team. R: A Language and Environment for Statistical Computing. *R Foundation for Statistical Computing* 2022.
Romero, P., *et al.* Computational prediction of human metabolic pathways from the complete human genome. *Genome Biology* 2005;6(1):R2.
Rosvall, M. and Bergstrom, C.T. Maps of random walks on complex networks reveal community structure. *Proceedings of the national academy of sciences* 2008;105(4):1118-1123.
Schaefer, C.F., *et al.* PID: the pathway interaction database. *Nucleic Acids Research* 2009;37(Database issue):D674-679.
Schauberger, P. and Walker, A. openxlsx: read, write and edit xlsx files (R package version 4.2.5.2). In.; 2023.
Shannon, P., *et al.* Cytoscape: a software environment for integrated models of biomolecular interaction networks. *Genome research* 2003;13(11):2498-2504.
Shen, W.-K., *et al.* AnimalTFDB 4.0: a comprehensive animal transcription factor database updated with variation and expression annotations. *Nucleic acids research* 2023;51(D1):D39-D45.
Sievert, C., Cheng, J. and Aden-Buie, G. 2023. bslib: Custom 'Bootstrap' 'Sass' Themes for 'shiny' and 'rmarkdown'. Release 0.5.1
Stöckel, D., *et al.* Multi-omics enrichment analysis using the GeneTrail2 web service. *Bioinformatics* 2016;32(10):1502-1508.
Szklarczyk, D., *et al.* STRING v11: protein–protein association networks with increased coverage, supporting functional discovery in genome-wide experimental datasets. *Nucleic acids research* 2019;47(D1):D607-D613.
Tomasoni, M., *et al.* MONET: a toolbox integrating top-performing methods for network modularization. *Bioinformatics* 2020;36(12):3920-3921.
Totu, T. 2024. NOODAI: Network Oriented multi-Omics Data Analysis and Integration. https://github.com/TotuTiberiu/NOODAI

Totu, T., *et al.* Delineation of signaling routes that underlie differences in macrophage phenotypic states. *bioRxiv* 2024:2024.2001. 2012.574349.
Urbanek, S. and Ts'o, T. 2022. uuid: Tools for Generating and Handling of UUIDs. Release 1.01.0
Van Eck, N.J. and Waltman, L. Visualizing bibliometric networks. In, *Measuring scholarly impact: Methods and practice*. Springer; 2014. p. 285-320.
Wickham, H. Reshaping Data with the reshape Package. *J Stat Softw* 2007;21(12).
Wickham, H. ggplot2: Elegant Graphics for Data Analysis,[computer program], 2016. In.: Springer-Verlag New York; 2020.
Wickham, H., *et al.* Package 'readxl'. 2023.
Wickham, H., *et al.* 2023. dplyr: A Grammar of Data Manipulation. Release 1.1.2
Wickham, H., *et al.* 2022. devtools: Tools to Make Developing R Packages Easier. Release 2.4.5
Wickham, H., *et al.* Package 'readr'. *Read Rectangular Text Data. Available online:* https://cran.r-project.org/web/packages/readr/readr.pdf 2023.
Wickham, H. and Wickham, M.H. Package 'stringr'. *Website:* http://stringr.tidyverse.org, https://github.com/tidyverse/stringr 2019.
Wu, T., *et al.* clusterProfiler 4.0: A universal enrichment tool for interpreting omics data. *The Innovation* 2021;2(3):100141.
Yamamoto, S., *et al.* INOH: ontology-based highly structured database of signal transduction pathways. *Database (Oxford)* 2011;2011:bar052.
Yu, H., *et al.* The importance of bottlenecks in protein networks: correlation with gene essentiality and expression dynamics. *PLoS computational biology* 2007;3(4):e59.
Zhou, G., Ewald, J. and Xia, J. OmicsAnalyst: a comprehensive web-based platform for visual analytics of multi-omics data. *Nucleic Acids Research* 2021;49(W1):W476-W482.
Zhou, G., *et al.* OmicsNet 2.0: a web-based platform for multi-omics integration and network visual analytics. *Nucleic Acids Research* 2022;50(W1):W527-W533.
